# Single ingestion of soy β-conglycinin induces increased postprandial circulating FGF21 levels exerting beneficial health effects

**DOI:** 10.1038/srep28183

**Published:** 2016-06-17

**Authors:** Tsutomu Hashidume, Asuka Kato, Tomohiro Tanaka, Shoko Miyoshi, Nobuyuki Itoh, Rieko Nakata, Hiroyasu Inoue, Akira Oikawa, Yuji Nakai, Makoto Shimizu, Jun Inoue, Ryuichiro Sato

**Affiliations:** 1 Department of Biotechnology, Graduate School of Agricultural and Life Sciences, The University of Tokyo, Tokyo 113-8657, Japan; 2 Institute of Gerontology, The University of Tokyo, Tokyo 113-8656, Japan; 3 Medical Innovation Center, Kyoto University Graduate School of Medicine, Sakyo, Kyoto 606-8507, Japan; 4 Department of Food Science and Nutrition, Nara Women’s University, Kita-Uoya-Nishi-Machi, Nara, 630–8506, Japan; 5 RIKEN Center for Sustainable Resource Science, Yokohama, Kanagawa, Japan; Faculty of Agriculture, Yamagata University, Tsuruoka-shi, Yamagata 997-8555, Japan; 6 Institute for Food Science, Hirosaki University, Aomori 038-0012, Japan

## Abstract

Soy protein β-conglycinin has serum lipid-lowering and anti-obesity effects. We showed that single ingestion of β-conglycinin after fasting alters gene expression in mouse liver. A sharp increase in fibroblast growth factor 21 (*FGF21*) gene expression, which is depressed by normal feeding, resulted in increased postprandial circulating FGF21 levels along with a significant decrease in adipose tissue weights. Most increases in gene expressions, including *FGF21*, were targets for the activating transcription factor 4 (ATF4), but not for peroxisome proliferator-activated receptor α. Overexpression of a dominant-negative form of ATF4 significantly reduced β-conglycinin-induced increases in hepatic *FGF21* gene expression. In *FGF21*-deficient mice, β-conglycinin effects were partially abolished. Methionine supplementation to the diet or primary hepatocyte culture medium demonstrated its importance for activating liver or hepatocyte ATF4-FGF21 signaling. Thus, dietary β-conglycinin intake can impact hepatic and systemic metabolism by increasing the postprandial circulating FGF21 levels.

Obesity increases susceptibility to insulin resistance that can lead to metabolic syndrome. Under obesity conditions, excess energy is stored as triglycerides in the liver as well as in adipose tissues, which worsens insulin sensitivity and causes hyperlipidemia. Soy protein, unlike animal proteins, is known to have beneficial effects by preventing body weight gain and improving blood lipid profiles^1^. The US Food and Drug Administration (FDA) authorized the health claim that daily ingestion of 25 g soy protein reduces the risk of cardiovascular heart disease^2^. Studies on the favorable effects of soy products have indicated that the effects are mostly exerted by the protein itself although isoflavones contained in an isolated soy protein fraction might make a minimal contribution to these effects^3^.

Soybeans contain abundant storage proteins and are highly valued as a nutritionally balanced dietary protein source. Soy protein is not a single protein but is composed of several types of proteins with different characteristics. Among these proteins, β-conglycinin, comprising approximately 20% of the total soy protein, has been studied with the idea that it might explain most of soy’s beneficial effects. A number of animal and human studies showed that β-conglycinin, when compared with milk protein (casein), has anti-atherosclerotic, serum triglyceride-reducing, cholesterol-lowering, and anti-obesity effects^4^,^5^,^6^. Therefore, the focus of this study was to characterize these favorable physiological responses produced by dietary β-conglycinin. It should be noted that dietary soy protein appears to alter gene expression in adipose tissue and in particular may have induced an increase in expression of the adiponectin gene (*Adipoq*) expression and circulating adiponectin levels^7^. Although increased circulating Adipoq may partially explain physiological responses driven by dietary β-conglycinin or soy protein, the molecular mechanism for increasing *Adipoq* expression remains unknown^8^.

Here we show that gene expression in mouse liver is altered following β-conglycinin consumption, accompanied by a decrease in blood glucose and insulin levels. The most significant increases were in fibroblast growth factor 21 gene (*FGF21*) expression in the liver and circulating FGF21 levels, which resulted in reduced body weight gain in response to a high-fat diet (HFD) intake. More surprisingly, a single ingestion of β-conglycinin after overnight fasting, rather than long-term feeding, was enough to induce an increase in hepatic *FGF21* gene expression and circulating postprandial FGF21 levels. A number of reports have demonstrated that dietary conditions such as fasting, protein restriction, and amino acid deprivation induce increased hepatic *FGF21* expression as observed in hepatocytes cultured with the amino acid deprivation media^9^,^10^,^11^,^12^,^13^,^14^,^15^,^16^. In this study, we first report that a rapid activation of activating transcription factor 4 (ATF4) following consumption of a β-conglycinin-containing diet (not a low-protein or amino acid-deficient diet) triggers a significant increase in *FGF21* expression in the liver, with a concurrent increase in lipolytic gene expression in epididymal white adipose tissue. We propose that a certain type of dietary protein have the ability to induce postprandial increases in serum FGF21 levels and exert beneficial health effects.

## Results

### Single ingestion of β-conglycinin after fasting causes a significant increase in hepatic FGF21 expression

**Figure 1:**
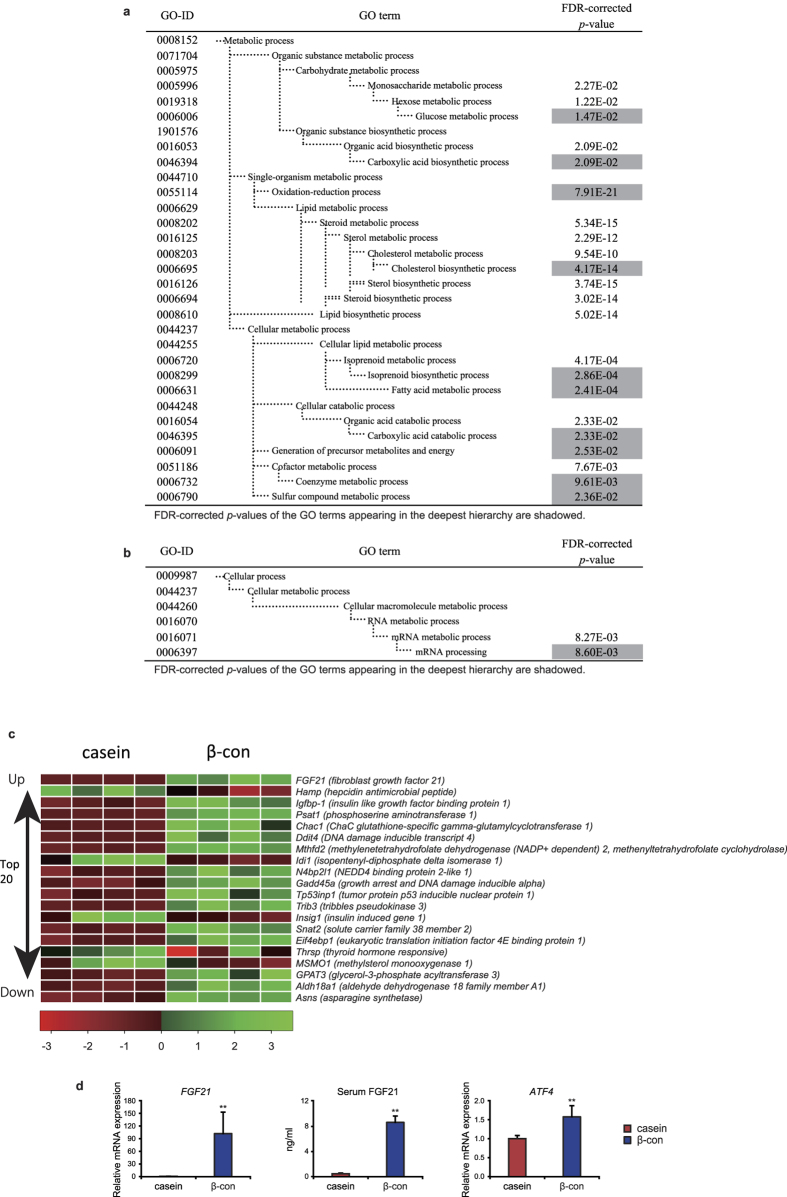
Hepatic gene expression following β-conglycinin consumption for 6 h after 24 h fasting. Five-week old male mice were acclimated to the casein high-fat diet for 3 days. After 24 h fasting, mice were fed with either the casein or β-conglycinin (β-con) high-fat diet for 6 h as shown in Supplementary Table 3. Using liver total RNA, microarray analysis was performed according to the manufacturer’s instructions (n = 4). (**a**) GO terms associated with the genes that were up-regulated in the β-conglycinin group. FDR-corrected *P* values were defined by the modified Fisher’s exact test with the Benjamini and Hochberg FDR correction. FDR-corrected *P* values < 0.05 are shaded in gray. (**b**) GO terms associated with the genes that were down-regulated in the β-conglycinin group. (**c**) Heatmap shows top twenty genes up-regulated or down-regulated by β-conglycinin ingestion from DNA microarray experiments. Green color indicates upregulation and red indicates downregulation in response to ingestion of two types of diet. (**d**) RT-qPCR was performed using total RNA used in DNA microarray experiments (n = 5). Relative expression levels of FGF21 and ATF4 mRNA in the liver are normalized to cyclophilin mRNA levels and are shown as fold induction to expression levels in the casein group. Serum FGF21 concentrations are determined by ELISA. All data are expressed as means ± SD (n = 5). **P < 0.01 determined by two-tailed Student’s *t*-test.

Previously published papers and our preliminary experiments showed that long-term consumption of β-conglycinin as a dietary protein source resulted in an improvement in lipid metabolism and prevented body weight gain in mice^5^,^8^,^17^. These findings raised the hypothesis that slight changes in gene expression, particularly in the liver, initiated by a single ingestion of β-conglycinin, rather than long-term consumption, appear to have occurred. To address this issue, we attempted to find rapid changes in gene expression using intensive DNA microarray analyses with hepatic RNA prepared from mice fasted for 24 h and then fed either a β-conglycinin- or casein-containing HFD for 6 h. To identify gene ontology (GO) terms that were overrepresented among the differentially expressed genes, we first performed a gene-annotation enrichment analysis using the online software program the Database for Annotation, Visualization, and Integrated Discovery (DAVID). The GO terms were significantly enriched in the genes that were up-regulated after β-conglycinin treatment are summarized in Fig. 1a. The hierarchical structure of GO that facilitated the identification of more specific GO terms appeared more imbedded in the hierarchy. The GO terms enriched in the genes up-regulated by the β-conglycinin treatment were glucose metabolic, carboxylic acid biosynthetic, oxidation-reduction process, cholesterol/isoprenoid biosynthetic, fatty acid metabolic, carboxylic acid catabolic, generation of precursor metabolites and energy, and coenzyme/sulfur-compound metabolic process. The GO term significantly enriched in the genes down-regulated after β-conglycinin treatment was mRNA processing (Fig. 1b). These results clearly show that β-conglycinin ingestion rapidly altered the mRNA levels of genes involved in some metabolic processes that are known to be affected by β-conglycinin long-term feeding. Surprisingly, the heat map listing the genes significantly changed by β-conglycinin showed *FGF21* was the most highly up-regulated gene, followed by *Igfbp-1* and *Psat1* (Fig. 1c). Indeed, circulating FGF21 levels were significantly increased 6 h after ingestion of the β-conglycinin diet, in conjunction with a significant increase in its mRNA levels as determined by the real-time PCR (qPCR) method (Fig. 1d). Another distinguishing feature of the results shown in the heat map was that most of the up-regulated genes were targets of the transcription factor, ATF4; these genes included *Psat1*, *Chac1, Gadd45a, Trib3*, *Snat2*, and *Asns* (Fig. 1c). Interestingly, *ATF4* gene expression was also increased by β-conglycinin consumption (Fig. 1d). Although ATF4 is known to become activated in response to endoplasmic reticulum (ER) stress, the current DNA microarray results showed no increase in the expression of ER stress genes, such as *Xbp1* and *Bip*, indicating that no ER stress occurred after β-conglycinin consumption. Although the *hepcidin* gene (*Hamp*) involved in systemic iron homeostasis was the most highly down-regulated gene by β-conglycinin (Fig. 1c), we found in pilot experiments that ATF4 was not a direct regulator for *Hamp* gene expression; the precise mechanism, however, for its reduced expression remains unclear. Another gene down-regulated by β-conglycinin was *Insig1.* A decrease in this protein would theoretically result in the activation of sterol regulatory element-binding proteins, thereby leading to an increase in the synthesis of cholesterol and fatty acids. This assumption contradicts the favorable effects of β-conglycinin on lipid metabolism improvements; this has been confirmed in a couple of animal experiments by long-term β-conglycinin feeding^5^,^8^,^17^, and it seems unlikely that a decrease in *Insig1* would be physiologically relevant *in vivo*.

### FGF21 contributes to a large portion of the effects of β-conglycinin

**Figure 2:**
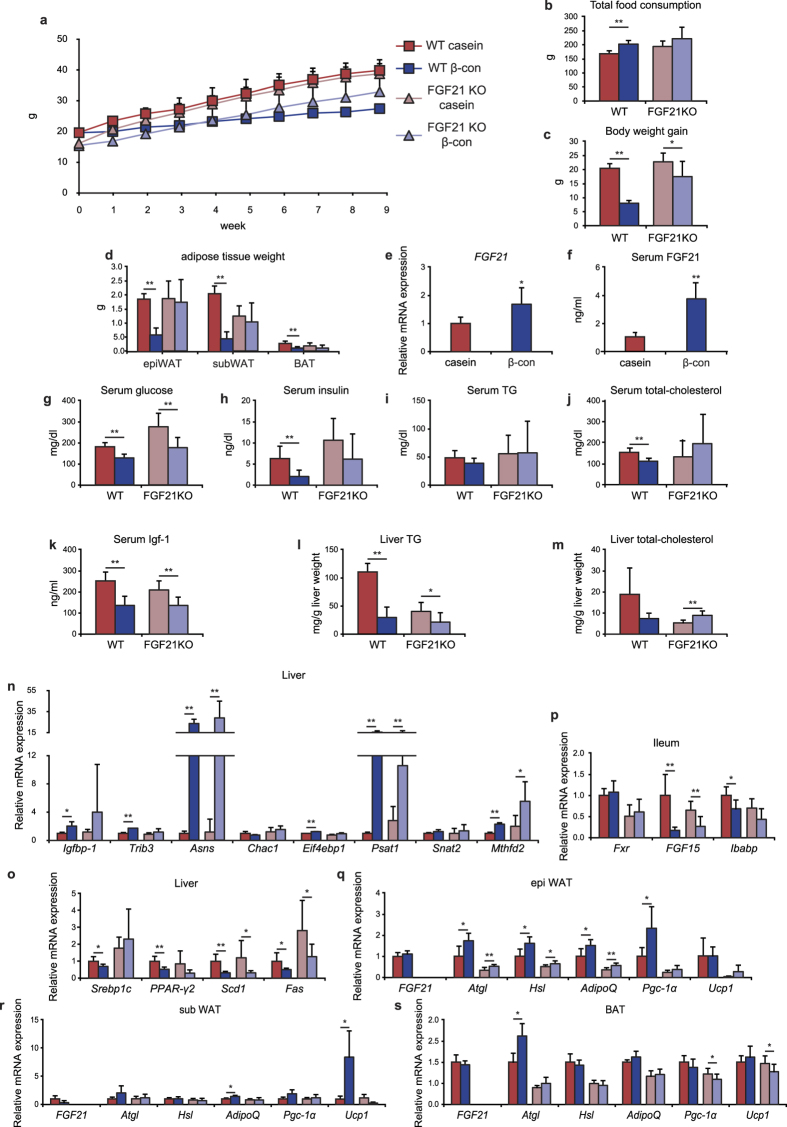
Beneficial effects of β-conglycinin in wild-type (WT) or FGF21-deficient (KO) mice. Five-week-old male WT and *FGF21* KO mice were acclimated to the casein high-fat diet for 7 days, and then fed with either the casein or β-conglycinin (β-con) diet for 9 weeks. After 6 h of starvation, mice were sacrificed. (**a**) Body weight of mice. All data are expressed as means ± SD (n = 6–8). (**b**) Total food consumption over 9 weeks. **P < 0.01 determined by two-tailed Student’s *t*-test. (**c**) Body weight gain over 9 weeks. (**d**) Adipose tissue (epididymal and subcutaneous white adipose tissue, and brown adipose tissue) weights of mice. (**e**) Expression levels of FGF21 mRNA in the liver are normalized to cyclophilin mRNA levels and are shown as fold induction to expression levels in the casein group of WT mice. *P < 0.05 determined by two-tailed Student’s *t*-test. (**f‒k**) Serum FGF21, glucose, insulin, triglyceride (TG), total cholesterol, and Igf-1 concentrations. (**l**) Liver TG contents. (**m**) Liver total cholesterol contents. (**n**) Expression levels of ATF4 target gene mRNA in the liver are normalized to cyclophilin mRNA levels and are shown as fold induction to expression levels in the casein group of WT mice. (**o**) Expression levels of mRNA related to lipid metabolism in the liver are normalized to cyclophilin mRNA levels and are shown as fold induction to expression levels in the casein group of WT mice. (**p**) Expression levels of mRNA related to bile acid metabolism in the ileum are normalized to β-actin mRNA levels and are shown as fold induction to expression levels in the casein group of WT mice. (**q‒s**) Expression levels of mRNA related to lipolysis in adipose tissues are normalized to 18S ribosomal RNA levels and are shown as fold induction to expression levels in the casein group of WT mice.

FGF21 is believed to be a humoral signal that increases insulin sensitivity and energy expenditure, thereby exerting multiple metabolic effects. Because DNA microarray analyses revealed a rapid increase in hepatic *FGF21* expression and circulating FGF21 levels following β-conglycinin ingestion, we aimed to determine whether increased circulating FGF21 levels remain elevated after a long-term β-conglycinin consumption, not just post- ingestion, and whether β-conglycinin still serves as a functional dietary protein in *FGF21*-deficient mice. Wild-type (WT) and *FGF21*-deficient mice were fed either a casein- or β-conglycinin-containing HFD for 9 weeks. Although β-conglycinin slightly prevented body weight gain even in *FGF21*-deficient mice, its anti-obesity effect was prominent in WT mice despite the fact that daily intake of the β-conglycinin diet was slightly but rather significantly higher, suggesting that FGF21 largely contributed to preventing body weight gain (Fig. 2a–c). Moreover, β-conglycinin significantly reduced adipose tissue weights (epididymal, subcutaneous white adipose tissue (WAT), and brown adipose tissue (BAT)), whereas no changes were observed in FGF21-deficient mice (Fig. 2d). Even though much time had passed after meal ingestion and the mice were fasted for 6 h, hepatic *FGF21* gene expression and serum FGF21 levels were significantly increased after the 9-week β-conglycinin feeding in WT mice (Fig. 2e,f), suggesting that increased circulating FGF21 might contribute to adipose tissue weight loss. Serum glucose levels were lowered by β-conglycinin in both types of mice, whereas serum insulin and total cholesterol levels were reduced only in WT mice (Fig. 2g,h,j). There was no significant change in serum triglyceride levels (Fig. 2i). Interestingly, serum levels of Igf-1, one of FGF21 target genes^18^, were decreased by β-conglycinin consumption in both types of mice, suggesting that the attenuation of body weight gain by β-conglycinin might be partly caused by this decrease (Fig. 2k). Liver triglyceride levels were lowered by β-conglycinin treatment more significantly in WT mice than those in the *FGF21*-KO mice (Fig. 2l). Liver total cholesterol levels underwent similar changes in serum levels in both types of mice (Fig. 2m). Hepatic gene expressions of other ATF4 targets, including *Asns* and *Psat1* that are involved in amino acid metabolism, were substantially increased by β-conglycinin in both WT and *FGF21*-deficient mice, suggesting that ATF4 served as the primary regulatory factor over *FGF21* (Fig. 2n). Consistent with previously published data^5^, β-conglycinin decreased the mRNA levels of *Fas* and *Scd1* in both types of mice, indicating that β-conglycinin effectively prevented fatty liver induced by HFD independently of FGF21 functions (Fig. 2o). Meanwhile, there were no significant changes in *PPAR*γ*2 and Srebp-1c* gene expressions induced by only β-conglycinin in *FGF21*-deficient mice; this was consistent with the finding that some serum profiles, including insulin, total cholesterol, and triglyceride concentrations, were not fully lowered by β-conglycinin consumption in these mice. When gene expressions in the ileum were analyzed, we found that β-conglycinin caused a decrease in gene expressions of Fxr targets, including *FGF15* and *Ibabp*, in both types of mice (Fig. 2p). These results suggest that β-conglycinin improved lipid metabolism through lowered bile acid absorption independently of FGF21.

A primary target of FGF21 is proposed to be adipose tissues, where it induces increased expression of a subset of genes related to energy expenditure. To address its action in adipose tissues in response to β-conglycinin consumption, we investigated the effect of the β-conglycinin diet on metabolic gene expression in epididymal, subcutaneous WAT, and BAT. In epididymal WAT, β-conglycinin consumption resulted in a significant increase in the expression of *Atgl*, *Hsl*, *Adipoq*, and *Pgc-1*α genes that have been reported to be up-regulated by FGF21 in several papers^19^,^20^,^21^,^22^. This increment was slightly observed in *FGF21*-deficient mice (Fig. 2q). The expression of Adipoq, Ucp-1 (in subcutaneous WAT) and Atgl (in BAT) was increased by β-conglycinin consumption (Fig. 2r,s). These results imply that the increased secretion of FGF21 from the liver in response to β-conglycinin intake caused loss of adipose tissue weights, accompanied by increased metabolic gene expression in WAT and BAT.

### Increased FGF21 gene expression in response to β-conglycinin ingestion is not caused by PPARα

**Figure 3:**
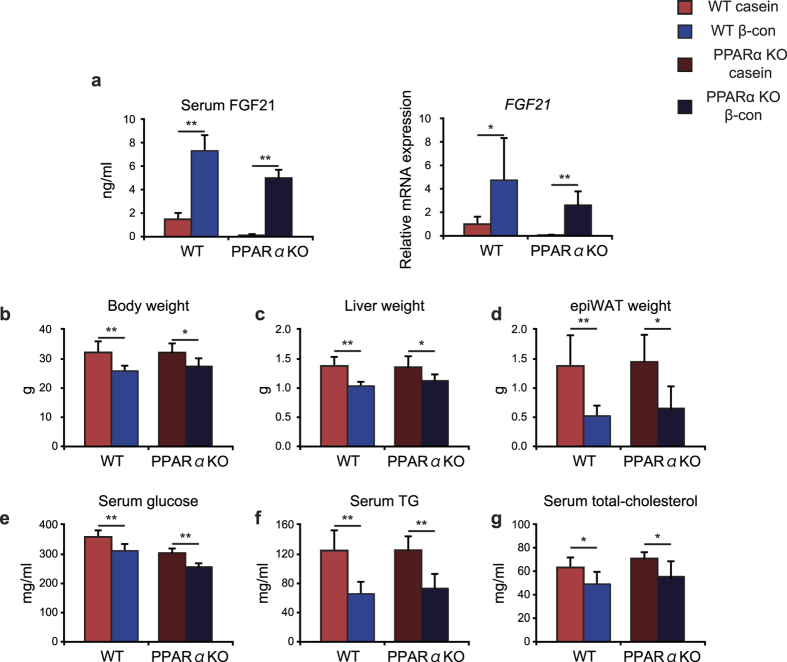
Beneficial effects of β-conglycinin in wild-type (WT) or PPARα-deficient (KO) mice. (**a**) Seven-week old male WT and PPARα KO mice were acclimated to the casein high-fat diet for 7 days, and then fed with either the casein or β-conglycinin (β-con) diet for 4 weeks. Expression levels of FGF21 mRNA in the liver are normalized to cyclophilin mRNA levels and are shown as fold induction to expression levels in the casein group of WT mice. Serum FGF21 concentrations are shown. All data are expressed as means ± SD (n = 5–6). **P < 0.01 determined by two-tailed Student’s *t*-test. (**b**) Body weight of mice. (**c**) Liver weight. (**d**) Epidydimal WAT weight. (**e**) Serum glucose concentrations. (**f**) Serum TG concentrations. (**g**) Serum total cholesterol concentrations.

*FGF21* expression is induced in the murine liver in response to fasting^18^,^19^. PPARα is believed to be a primary hepatic regulator of *FGF21* expression. Following food intake, the expression is likely to be reduced in conjunction with inactivation of PPARα. To investigate the effect of PPARα on increased *FGF21* expression by β-conglycinin consumption, WT and PPARα-deficient mice were fed either the casein- or β-conglycinin-containing diet for 4 weeks. We found that both hepatic *FGF21* expression and serum FGF21 levels became elevated even in PPARα-deficient mice (Fig. 3a). These results clearly show that there was no association between β-conglycinin ingestion and PPARα activation. The body, liver, and epididymal WAT weights were all reduced by β-conglycinin consumption in both types of mice (Fig. 3b–d). The β-conglycinin diet lowered serum glucose, TG, and total-cholesterol levels independently of PPARα (Fig. 3e–g). Taken together, we concluded that the favorable effects of β-conglycinin were not caused by activation of PPARα.

### ATF4 is involved in induction of increased FGF21 gene expression after β-conglycinin ingestion

**Figure 4:**
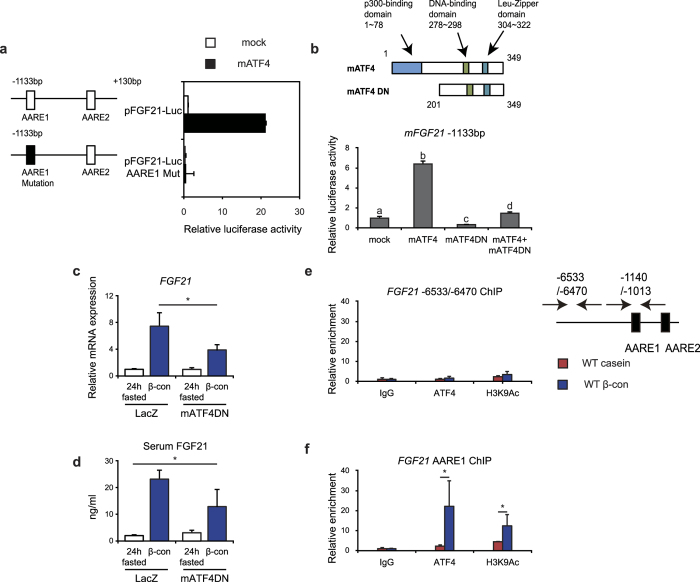
ATF4 stimulates hepatic *FGF21* gene expression following β-conglycinin ingestion. (**a**) Mouse primary hepatocytes were transfected with 200 ng of one of reporter constructs, either pCMV 3xFLAG (mock) or pCMV 3xFLAG mATF4, and a β-galactosidase construct. After incubation for 48 h, luciferase and β-galactosidase activities were determined. Normalized luciferase values were determined by dividing the luciferase activity by the β-galactosidase activity. Relative activities are shown as fold induction to expression levels in the mFGF21-1133 bp together with pCMV 3xFLAG. (**b**) HEK293T cells were transfected as described in Fig. 4a. In the fourth group the cells were transfected with 350 ng of pCMV 3xFLAG mATF4 and 515 ng of pCMV 3xFLAG mATF4DN (a dominant negative form of mATF4). The structure of mATF4 and mATF4DN are depicted. Relative luciferase activities are shown as fold induction to expression levels in the mFGF21-1133 bp together with pCMV 3xFLAG. One-way analysis of variance followed by the Bonferroni procedure was used. Different superscript letters denote statistical significance (*P* < 0.05). (**c**,**d**) Eight-week-old mice were injected through the tail vein with 1 × 10^9^ pfu of adenovirus bearing either Lac Z or mATF4DN. After 24 h, these mice were fasted for 24 h and then re-fed the β-con diet for 6 h. Hepatic FGF21 mRNA levels are normalized to 18S rRNA levels and are shown as fold induction to expression levels in the 24 h-fasted Lac Z group. Serum FGF21 concentrations are also shown. **P < 0.01 determined by two-tailed Student’s *t*-test. (**e**,**f**) Livers obtained from mice fed for 3 h were processed for ChIP analyses. After immunoprecipitation with anti-ATF4, anti-acetyl-histone H3, or control IgG, real-time PCR analysis was performed with a primer set covering the region (−6533 to −6470) of the promoter in the mouse *FGF21* gene, or the region covering the AARE sequence (as illustrated to the right side of Fig. 4e). The results were represented by fold enrichment relative to control IgG. The same results were obtained in two separate experiments. *P < 0.05 by two-tailed Student’s *t*-test. All data are expressed as means ± SD (n = 3).

We next investigated the effect of ATF4 on the *FGF21*’s promoter activity. Reporter gene analyses, using a luciferase construct with the 1.1-kb promoter region of the mouse *FGF21* gene, revealed that overexpression of mouse ATF4 enhanced its transcription through the upstream amino acid response element (AARE) 1 (Fig. 4a), one of two AAREs located in this region; this finding was consistent with previously published data^16^. In order to further confirm the involvement of ATF4 in elevation of *FGF21* expression, we examined whether a dominant negative form of ATF4 (mATF4DN)^23^, which consisted of the C-terminal half containing the basic-leucine-zipper domain but lacking the N-terminal 200 amino acid residues, hindered the stimulating effect of WT ATF4 on *FGF21* promoter activity. Overexpression of mATF4DN significantly reduced the increase in WT ATF4-induced luciferase activity (Fig. 4b). Based on these findings, mice were infected with either a control Lac Z or mATF4DN-containing adenovirus to determine whether the increased hepatic *FGF21* expression in response to β-conglycinin ingestion was hindered by overexpression of mATF4DN *in vivo*. β-conglycinin ingestion for 6 h after a 24 h fasting period significantly increased hepatic *FGF21* expression and circulating FGF21 levels in control mice; in contrast overexpressed *mAtf4dn* significantly lowered the increase in hepatic *FGF21* expression and serum FGF21 levels induced by β-conglycinin ingestion. These results indicated that the activation of ATF4 contributed to an increase in *FGF21* expression (Fig. 4c,d). Moreover, we examined whether increased ATF4 interacted with the AARE1 sequence on the genomic DNA in response to β-conglycinin ingestion. Using liver genomic DNA, ChIP assays were performed with either an anti-ATF4 or anti-acetylated histone 3 antibody. Whereas ATF4 interactions with the distal unrelated region was not observed in either diet (Fig. 4e), β-conglycinin ingestion induced an increased interaction of ATF4, together with acetylated histone 3, to the region containing the AARE1 sequence in the *FGF21* promoter region (Fig. 4f). When these results are combined, it is likely that ATF4 directly caused an increase in hepatic *FGF21* gene expression and serum FGF21 serum levels after β-conglycinin ingestion.

### Serum methionine levels control hepatic FGF21 gene expression

**Figure 5:**
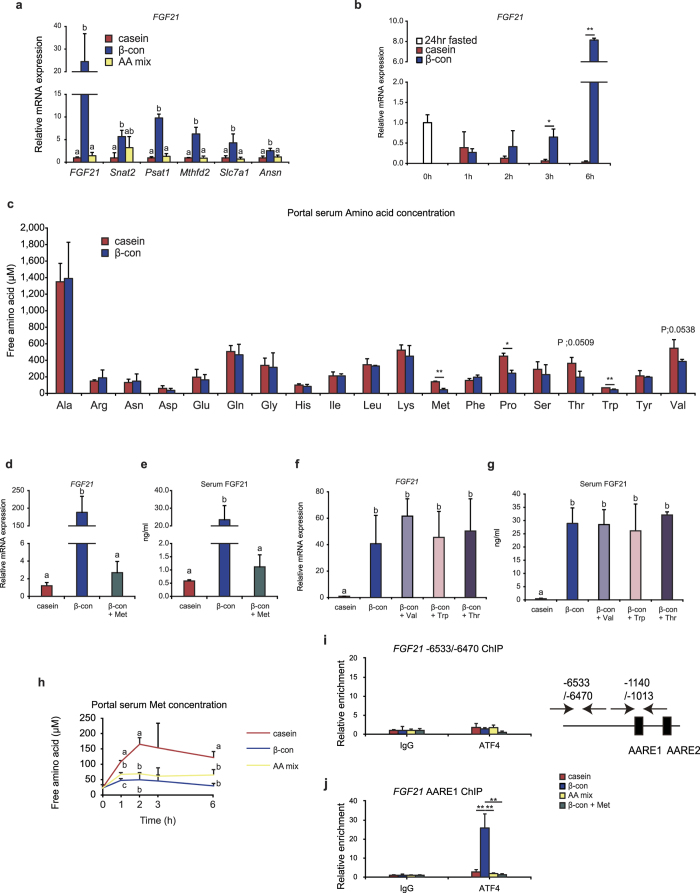
Methionine supplementation to the β-conglycinin diet negates the FGF21-stimulating effect. Five-week old mice were fasted for 24 h and then fed with the indicated high-fat diet. Using liver total RNA, RT-qPCR was performed and relative mRNA levels are normalized to cyclophilin mRNA levels. (**a**) Mice were fed with the diet for 6 h. Relative mRNA expression levels are shown as fold induction to expression levels of the indicated genes in the casein group. All data are expressed as means ± SD (n = 6). One-way analysis of variance followed by the Bonferroni procedure was used. Different superscript letters denote statistical significance (*P* < 0.05). (**b**) Mice fed with 1.2 g the diet and then sacrificed at the indicated time. Relative mRNA expression levels are shown as fold induction to expression levels at 0 (24-h fasted). All data (**b–i**) are expressed as means ± SD (n = 3). *P < 0.05 and **P < 0.01 determined by two-tailed Student’s *t*-test. (**c**) Portal blood serum was obtained at 1 h after feeding of the diet. (**d**,**e**) Mice were fed for 6 h. Relative mRNA expression levels are shown as fold induction to expression levels in the casein group. Serum FGF21 concentrations are also shown. Different superscript letters denote statistical significance (*P* < 0.05). (**f**,**g**) Mice were fed for 6 h. Relative mRNA expression levels are shown as fold induction to expression levels in the casein group. Serum FGF21 concentrations are also shown. Different superscript letters denote statistical significance (*P* < 0.05). (**h**) Mice were fed with 1.2 g of the diet and then portal blood was collected at the indicated time. Serum methionine concentrations are shown. Different superscript letters denote statistical significance (*P* < 0.05). (**i**,**j**) ChIP analyses using anti-ATF4 or control IgG were performed as described in Fig. 4e,f. The results were represented by fold enrichment relative to control IgG in 4 different groups. The same results were obtained in two separate experiments.

There is a difference in amino acid composition between milk protein casein and soy protein β-conglycinin. In particular, methionine content in β-conglycinin corresponds to approximately one-third of that found in casein. We, therefore, examined whether a newly designed diet composed of the mixture of amino acids included in β-conglycinin has the same postprandial effect as the whole β-conglycinin diet. Hepatic expression of ATF4 target genes, including *FGF21*, was greatly elevated after 6 h feeding in mice fed with only the β-conglycinin diet, whereas the amino acid mixture diet had no effect in a manner similar to the casein diet (Fig. 5a). These results indicate no causal relationship between the amino acid composition of β-conglycinin and the induced activation of the ATF4-FGF21 signaling axis. We next examined when *FGF21* transcription started to increase after the beginning of β-conglycinin ingestion. In preliminary experiments, we confirmed that mice ate at least 1.2 g of one of the diets within 1 h after 24 h fasting. Mice were fed with 1.2 g of one of the experimental diets for 1 h after 24 h fasting and were then sacrificed at 1, 2, 3, and 6 h after the initiation of feeding. After 24 h fasting *FGF21* expression was significantly increased, but at 1 or 2 h after feeding of any of the diets *FGF21* expression levels were lower than those at 0-h level (Fig. 5b). This is most likely due to postprandial inactivation of PPARα that was significantly activated in response to the 24 h fasting. After 2 to 6 h, casein feeding further lowered *FGF21* expression. In contrast, after 3 to 6 h, *FGF21* expression was greatly increased in mice fed with the β-conglycinin diet, and at 6 h the expression level greatly exceeded the initial 0 time level. These results imply that liver recognized certain signals within 3 h after β-conglycinin ingestion.

Based on these results, we sought to determine free amino acid concentrations in portal blood serum at 1 h after feeding of either the casein or β-conglycinin diets. There were no significant differences in overall free amino acid concentrations between two groups (Fig. 5c). However, among essential amino acids we found decreased concentrations of methionine, threonine, tryptophan, and valine in the β-conglycinin-fed mice. Several groups reported that treatment with a methionine-choline-deficient diet increased serum FGF21 levels despite induction of non-alcoholic steatohepatitis^24^,^25^. Based on these reports, we first examined whether methionine supplementation affected the increase in hepatic *FGF21* expression and circulating FGF21 levels induced by β-conglycinin. Mice were fed with the methionine-supplemented diet, which was prepared by adding enough methionine to achieve a twofold increase in its dietary content (Supplementary Table 1), for 6 h after 24 h fasting. We found that the β-conglycinin effect was almost completely eliminated by this supplementation (Fig. 5d,e). In contrast, supplementation of one of three other amino acids had no effect on reducing the increased *FGF21* expression and serum FGF21 levels caused by β-conglycinin consumption (Fig. 5f,g). These results imply that insufficient methionine availability, occurring only when mice were fed with β-conglycinin, resulted in the postprandial increase in hepatic *FGF21* expression and serum FGF21 levels. If this was the case, the increased ATF4 protein should have been detected in the liver within 3 h. Western blot analysis revealed that at 2 h after β-conglycinin ingestion, hepatic ATF4 protein increased when compared with casein (Supplementary Fig. 1). However, an increase in ATF4 protein was also observed in the livers of mice fed either the amino acid mixture or the methionine-supplemented diet despite lack of elevation of *FGF21* transcription caused by these diets. Moreover, an upstream regulatory factor for ATF4, phosphorylated eukaryotic initiation factor 2α (p-eIF2 α), was not increased in response to feeding of any kind of diet. Taken together, it seems likely that ATF4 protein levels were not necessarily a determinant for promoting ATF4’s target gene expression in response to β-conglycinin ingestion. Furthermore, we repeatedly examined time-dependent changes in free methionine concentration in the portal blood serum for 6 h after ingestion of several types of diets. Among the three diets, the β-conglycinin diet kept the methionine concentration at the lowest level during the 6 h period (Fig. 5h). In particular, a significant difference between the amino acid mixture and β-conglycinin diet was observed 1 h after feeding, suggesting that lowered serum methionine just after ingestion of β-conglycinin might result in a change in liver *FGF21* expression.

To verify that decreased ATF4 interacted with the AARE1 sequence on the genomic DNA in response to ingestion of the methionine-supplemented or amino acid mixture diet, ChIP assays were performed with an anti-ATF4 antibody as shown in Fig. 4e. β-conglycinin ingestion again induced an increased interaction of ATF4 to the region containing the AARE1 sequence in the FGF21 promoter region, whereas this increase was not observed after ingestion of other 3 diets (Fig. 5I,j).

### FGF21 expression is increased in hepatocytes cultured in a medium with a lower methionine concentration

**Figure 6:**
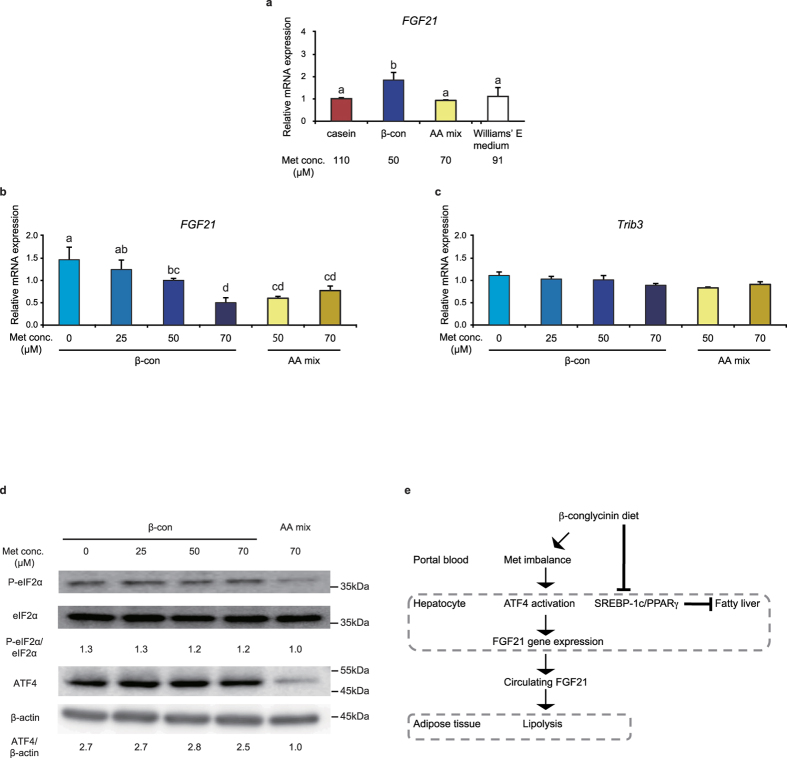
Induced FGF21 gene expression in hepatocytes exposed to the media containing varying levels of methionine. Mouse primary hepatocytes were cultured with an amino acid-deficient medium for 1 h followed by the indicated medium for 2 h. Using their total hepatocyte RNA, RT-qPCR was performed and relative mRNA levels were normalized to 18 S ribosomal RNA levels. (**a**) Hepatocytes were cultured with the medium in which the amino acid concentrations were set up comparable with those in the portal blood serum of mice fed with one of the diets (the casein, β-con, or amino acid mixture) or Williams’ E medium. All data are expressed as means ± SD (n = 3). One-way analysis of variance followed by the Bonferroni procedure was used. Different superscript letters denote statistical significance (*P* < 0.05). (**b**,**c**) Hepatocytes were cultured with either the β-con or AA mix medium with various concentrations of methionine for 2 h. All data are expressed as means ± SD (n = 3). Different superscript letters denote statistical significance (*P* < 0.05). (**d**) Western blot analysis of the indicated protein expression in the cells cultured with the medium containing various concentrations of methionine for 2 h. β-actin serves as a loading control. The same results were obtained in three separate experiments. (**e**) Schematic model of the upregulation of hepatic *FGF21* gene expression caused by β-conglycinin consumption.

We hypothesized that the increase in hepatic *FGF21* expression at 3 h after β-conglycinin ingestion might be triggered by a decrease in methionine concentration in the portal blood at 1 h. To assess this possibility, primary mouse hepatocytes were cultured in medium that contained free amino acid concentrations equal to those found in the portal blood at 1 h after ingestion of each diet (Supplementary Table 2). The total amino acid concentrations in Williams’ E medium (6.6 mM) are close to those in the postprandial portal blood at 1 h after ingestion of the casein, β-conglycinin, or amino acid mixture diet (4.7‒5.7 mM). Interestingly, the culture medium mimicking the amino acid concentrations in the portal blood of mice fed with the β-conglycinin diet induced an increase in *FGF21* expression; in contrast, the culture media mimicking those for the amino acid mixture or methionine-supplemented diet underwent no changes in *FGF21* expression when compared with the casein and Williams’ E medium (Fig. 6a). Incremental increases in methionine concentrations starting at 50 μM, which corresponded to its concentration in portal blood of mice fed with the β-conglycinin diet, to 70 μM, which corresponded to that for the amino acid mixture diet, significantly reduced *FGF21* expression (Fig. 6b and Supplementary Table 2). In contrast, a decrease in methionine concentration in the β-conglycinin medium increased *FGF21* expression in a dose-dependent manner. Furthermore, a decrease in methionine concentration in the amino acid mixture from 70 to 50 μM had no effect on *FGF21* expression even though the methionine concentration was reduced to the same level as that in the β-conglycinin medium; these results suggest that the methionine concentration itself is not a determinant for regulating *FGF21* transcription. *Trib3*, another ATF4 target gene, showed a similar the dose-response pattern, but there were no significant differences (Fig. 6c). To examine molecular changes of the upstream factors involved in an increase in *FGF21* expression, western blot analysis was performed for detecting phosphorylated eIF2α and ATF4 proteins. Both of them were increased in hepatocytes cultured with the β-conglycinin medium independent of the methionine concentration when compared with the amino acid mixture medium (Fig. 6d). When these results are taken together, it appears that hepatocytes might function in sensing the methionine imbalance in the culture medium by accentuating *FGF21* transcription via activation of ATF4. The same phenomenon may happen in the liver following ingestion of β-conglycinin.

## Discussion

The present study is the first to describe the molecular mechanism(s) by which ingestion of β-conglycinin, one of the soy proteins, has beneficial health effects. Notably, an unexpected finding was that the single ingestion of β-conglycinin induced increased hepatic *FGF21* expression and postprandial FGF21 circulating serum levels; this was in sharp contrast with a control dietary protein (casein), which severely reduced both *FGF21* expression and protein levels. It is usually accepted that the quality of dietary proteins can be evaluated after long-term feeding on the basis of the notion that their physiological effects are produced slowly over time. Nevertheless, DNA microarray analyses performed in the present study enabled us to detect rapid changes in gene expression caused by a single ingestion of different types of dietary proteins. Importantly, even after long-term feeding of β-conglycinin (up to 9 weeks) we found that the increase in hepatic *FGF21* expression and circulating FGF21 levels remained in a steady state, resulting in prevention of body weight gain, a decrease in blood glucose and liver triglyceride levels, and weight loss of adipose tissues (Fig. 6e).

We also found that a single ingestion of β-conglycinin significantly induced an increase in gene expressions, including *FGF21*, which are regulated by the transcription factor, ATF4, and have been shown to become active in response to ER stress (Fig. 1b). Because no significant increases in ER stress marker genes were observed, it seems obvious that β-conglycinin ingestion activated ATF4 with no direct ER stress involvement. Our present findings indicated that hepatic adenovirus-mediated expression of a dominant form of ATF4 reduced β-conglycinin-induced *FGF21* expression and that a larger amount of ATF4 protein was recruited to the AARE motif localized in the *FGF21* promoter region. We then wondered what was a determinant of ATF4 activation caused by the different dietary proteins. It is also known that dietary protein deficiency or an imbalance of essential amino acids increases ATF4 synthesis^12^,^13^,^16^,^26^. Mechanistically, the general control non-depressible 2 (GCN2) kinase acts as an amino acid sensor, leading to phsophorylation of the transcriptional initiation factor (eIF) 2α that suppresses general protein synthesis, but promotes an increase in translation of ATF4 mRNA. Several previously published papers showed a significant increase in ATF4 protein levels in cultured cells in response to an imbalance of essential amino acids, but only a slight change in its protein levels *in vivo*, with some exceptions, in response to either dietary protein deficiency or amino acid imbalance; these results are consistent with our present results^10^,^13^,^27^,^28^. Because *FGF21* expression significantly increased after a single ingestion of β-conglycinin in the current study, we predicted a sharp increase in ATF4 protein levels, but failed to detect it. At this time we do not know how ATF4 activity is induced. It is tempting to speculate that ATF4 protein modification such as phosphorylation or acetylation might occur and promote the transcriptional activation in response to β-conglycinin consumption^29^,^30^,^31^. Alternatively, we cannot rule out the possibility that other molecules capable of interacting with and modulating ATF4 might accelerate the transcription of ATF4 target genes^32^. Further studies will be required to determine the mechanism for liver ATF4 activation in response to β-conglycinin ingestion.

The finding that the amino acid mixture diet failed to reproduce the effect caused by β-conglycinin ingestion implies the possibility that certain peptides derived from digestion of β-conglycinin exerted a pharmacological action. However, this hypothesis was contradicted by the finding that the culture medium amino acid composition for primary hepatocytes played a critical role in increasing *FGF21* expression. It is interesting that the transient methionine imbalance in portal blood occurred after β-conglycinin ingestion might result in an increase in hepatic *FGF21* expression and circulating FGF21 levels. β-conglycinin is composed of three types of subunits, α, α′, and β with all of the subunits sharing an identical N-terminal amino acid sequence (methionine-methionine-arginine). As a result, these six methionine residues among 11 residues contained in the three subunits led to disproportionate localization in their N-terminus. While β-conglycinin was digested and absorbed in the small intestine, this methionine dipeptide, if generated during the digestive process, might have a causal influence on the lower concentrations of free methionine in portal blood just after ingestion of β-conglycinin when compared with ingestion of the free amino acid mixture. Another unexpected finding in the current study was that primary mouse hepatocytes showed an increase in *FGF21* expression when incubated with medium that contained identical amino acids to those observed in portal blood of mice fed with β-conglycinin when compared with those receiving casein. It is important to note that the low methionine concentration itself did not trigger increased *FGF21* expression, based on the finding that the reduction of methionine concentrations in the culture medium to the same level as that for β-conglycinin failed to increase *FGF21* expression (Fig. 6b). Nevertheless, the finding that incrementally reducing methionine concentration down to zero resulted in a further increase in *FGF21* expression in cultured hepatocytes (Fig. 6b) shows the importance of methionine; these findings are consistent with previously published findings^12^,^26^. It appears that a certain balance ratio between methionine and other amino acids might be critical for causing metabolic responses in hepatocytes. The same phenomenon was also shown in a report in which reduced alanine concentration in the culture medium of primary hepatocytes resulted in increased *FGF21* expression^13^. In the current study, we observed no changes in portal blood alanine concentrations when mice were fed either the casein or β-conglycinin diet (Fig. 5c). Meanwhile, β-conglycinin diet supplementation with one of essential amino acids, which were lowered in portal blood of mice fed with β-conglycinin, had no effect on increased hepatic *FGF21* expression with the exception of methionine supplementation (Fig. 5). These findings suggest the particularity of methionine. These results also indicate that the methionine imbalance in portal blood might directly or indirectly elicit the ATF4-FGF21 signaling axis activation in hepatocytes. Further studies are needed to elucidate the precise mechanism for sensing the methionine imbalance by hepatocytes.

It should be noted that an increase in circulating FGF21 in response to 9-week β-conglycinin consumption resulted in induction of lipolytic (*Hsl* and *Atgl*), *Pgc-1*α, and *Adipoq* expressions in adipose tissues in an FGF21-dependent manner (Fig. 2). In addition, we observed an increase in the Ucp-1 mRNA and Pgc-1α protein levels in subcutaneous WAT of WT mouse fed with β-conglycinin, but not of FGF21-deficient mouse (Fig. 2r and Supplementary Fig. 2), suggesting that β-conglycinin might induce browning of subcutaneous WAT. These increases appear to contribute to a decrease in body weight gain and adipose tissue weights, and serum glucose levels. In a pilot experiment for 9 weeks, we observed increased energy expenditure in β-conglycinin-fed mice without any significant increase in locomotor activity (Supplementary Fig. 3). Although an increase in Ucp-1 expression is believed to assist browning of WAT, recent papers demonstrated that the effects of FGF21 on body weight reduction and glucose homeostasis can occur in Ucp-1 knockout mice^33^,^34^. In contrast, some authors claim that FGF21 requires UCP1 to improve glucose clearance, likely involving increased UCP1-dependent thermogenesis^35^. Further experiments will be required to elucidate the link between FGF21 and Ucp-1 involved in beneficial effects caused by β-conglycinin.

Adiponectin, an adipokine predominantly secreted from adipocytes, regulates glucose and lipid metabolism and improve insulin sensitivity. Because FGF21 enhances both expression and secretion of adiponectin in adipocytes^36^, elevation of circulating adiponectin levels on the basis of increased Adipoq gene expression in epididymal and subcutaneous WAT might be implicated in favorable effects caused by β-conglycinin consumption. Contrary to our expectation, no significant difference in the adiponectin levels was observed in both WT and FGF21-deficient mice (supplementary Fig. 4). It seems likely that the increase in Adipoq gene expression was not enough to induce increased its circulating levels.

Soy proteins have gained considerable attention for their role in improving risk factors for cardiovascular disease. In 1999, the FDA approved labeling for foods containing soy proteins as protective against coronary heart disease^2^. In contrast, the American Heart Association Nutrition Committee recently released a scientific advisory on soy protein, isoflavones, and cardiovascular health showing such favorable effects had not been confirmed^37^. Therefore, it is still controversial whether soy protein intake exhibits beneficial health effects in humans. Nevertheless, this study demonstrated that one of the soy protein components, β-conglycinin, produced a large positive metabolic impact in mice. So far several reports have shown that plasma FGF21 levels are not consistently increased by fasting in humans unlike in rodents, clarifying a difference in controlled induction between species^38^,^39^,^40^. Moreover, because consuming a single protein in a normal eating routine is totally impractical in humans, it is hard to apply the current findings observed in rodents to humans. However, even in humans, it might theoretically be achievable to design dietary protein intake for exerting metabolic effects through activation of the ATF4-FGF21 pathway. This could be done by monitoring subtle alterations in portal blood amino acid levels after meal ingestion and controlling them. Our current study sheds light on the postprandial impact of dietary protein intake on health and metabolism.

## Methods

### Plasmids

The mouse ATF4 (mATF4) construct was synthesized from total mouse hepatocyte RNA and cloned into the p3x FLAG-CMV-7 vector (Sigma-Aldrich Co.) using EcoRI and BamH1 sites. A mouse ATF4 dominant negative (mATF4 DN) construct was synthesized from the mATF4 construct. For adenoviral experiments, a mATF4 DN vector was subcloned into the corresponding sites of the Gateway pENTR1A vector (Invitrogen) and recombined into the destination vector pAd/CMV/V5-DEST (Invitrogen).

### Mice and Diets

All experiments were performed with male mice. C57BL6 mice were obtained from CLEA Japan Inc. PPAR α −/− was obtained from Jackson laboratory. FGF21 −/− mice were kindly provided by Dr. N Ito^41^. All animal experiments were approved by the animal experiment committee of the University of Tokyo and the Nara Women’s University and performed in accordance with the relevant guidelines and regulations. Mice were housed in a 12-h light/dark cycle, with the dark cycle occurring between 9:00AM and 9:00PM. The mice were given free access to water and were initially fed a standard pellet diet (Labo MR Stock; Nosan Corporation Bio Department) during a 7-day acclimation period. For the fasting and re-feeding experiments, 24 h-fasted mice were fed each diet for 6 h. The fasted and re-fed mice were sacrificed at ZT1 and ZT7, respectively. In log-term feeding experiments anesthetized mice were sacrificed after 6 h-fasting (ZT7 in Fig. 2) or at ZT2 without fasting as shown in Fig. 3. The livers, intestines, and adipose tissues were rapidly excised, frozen in liquid nitrogen, and stored at −80 °C until further processing. Blood samples were obtained, and the serum was separated and stored at −80 °C until further processing. Diet composition is described in Supplementary Table 3.

### Metabolite Measurements

Serum Glucose concentrations were measured using Glucose C2 test (Wako Chemicals Inc.). Total hepatic lipids were extracted from an approximately 200 mg piece of frozen liver tissue as previously described^42^. Serum triglyceride concentrations and triglyceride content of liver were measured using triglyceride E-test wako (Wako Chemicals Inc.). Serum cholesterol concentrations and cholesterol content of liver were measured using cholesterol E-test wako (Wako Chemicals Inc.). Serum insulin concentrations were measured using Lbis^®^ Insulin-mouse ELISA Kit (T type) (SHIBAYAGI Co. Ltd.). Serum FGF21 concentrations were measured using Mouse/Rat FGF21 Quantikine ELISA kit (R&D Systems, Inc.). Serum Igf-1 concentrations were measured using Mouse/Rat IGF-1 Quantikine ELISA kit (R&D Systems, Inc.).

### Amino acid analysis

Frozen tissue and serum samples were homogenized using a Precellys^®^ 24 homogenizer (Bertin Technologies, Montigny le bretonneux, France) in a cold 80% methanol solution containing L-phenyl-d5-alanine as an internal standard and partitioned with chloroform to remove hydrophobic components such as lipids. The water-soluble fractions were concentrated 10-fold using a centrifugal evaporator and applied for the amino acid analysis. The determinations of amino acids were performed according to the recently established method of LC-MS/MS^43^,^44^,^45^.

### Mouse primary hepatocyte in culture

Mice primary hepatocytes were isolated from mouse liver by the two-step collagenase perfusion method. Briefly, Hank’s buffered salt solution (Sigma-Aldrich Co.) followed by collagenase (Wako) digestion medium was perfused through a cannula inserted from superior vena cava. After the viability was checked, 6 × 10^5^ cells were plated in a collagen coated 6 well dish (Corning, USA) in Williams’ E Medium (Sigma-Aldrich Co.) supplemented with 10% fetal bovine serum, 10 nM dexamethasone, 2 mM glutamine and 100 units/ml penicillin, 100 μg/ml streptomycin and maintained at under 5% CO_2_ atmosphere.

### Luciferase Assay

The *FGF21* promoter construct −1133/+130 was generated by PCR using mouse genomic DNA and the following oligonucleotides: −1133 forward, 5′-ATATGAGCTCCAGGAAACAACCCAGCTCTT-3′; +130 reverse, 5′-ATATCTCGAG AGGCAGCTGGAATTGTGTTC-3′. The PCR-amplified fragments were cloned into a luciferase reporter construct using Sac I and Xho I sites. To generate the AARE mutation fragment, −1133/+130 FGF21 promoter constructs and the following oligonucleotides were used: forward 5′-AGCGCAACGGGAGGACAGCAGCTGAGCACAA-3′; reverse 5′-TCCTAGTGCTTCTTTCACCAGACAG-3′.All constructs were verified by DNA sequencing.

For transient transfection assays in Fig. 4a, mice primary hepatocytes were obtained from twelve-week old C57BL/6 male mice after collagenase perfusion. Mice primary hepatocytes were maintained in Williams’ E medium containing 10% fetal bovine serum, 10 nM dexamethasone, 2 mM L-glutamine and 100 units/ml penicillin, and 100 μg/ml streptomycin and maintained under 5% CO_2._ Cells were plated in collagen coated 12-well plates at a density of 600,000 cells per well. For transient transfection assays in Fig. 4b, HEK293T cells were placed in 12-well plates at a density of 100,000 cells per well. These cells were maintained in DMEM supplemented with 10% fetal bovine serum, containing 100 units/ml penicillin, and 100 μg/ml streptomycin and maintained under 5% CO_2._ After 6hr, cells were transfected using Lipofectamine 2000 (Invitrogen). Each well was transfected with *FGF21* reporter gene, pCMV 3xFLAG or pCMV 3xFLAG mATF4, pCMV 3xFLAG mATF4 DN, and pCMV β-galactosidase. After 48hr transfection, luciferase and β-galactosidase assays were performed on Mini Lumat LB9506 (BERTHOLD). Luciferase activity was normalized to β-galactosidase activity.

### Western blot analysis

Approximately 100 mg of liver was homogenized in 1 ml lysis buffer (50 mM Tris/HCl, pH 8.0; 150 mM NaCl, 0.1% SDS, 0.5% deoxycholate, and 1% Triton X-100) using a Polytron homogenizer (Qiagen). After centrifugation at 16,000 × g for 5 min at 4 °C, the supernatant was collected and concentrations were measured using BCA kit (Thermo Fisher Scientific Inc). Western blotting was performed using antibodies against ATF4, p-eIf2α and total-eIF2α (Cell Signaling). Anti-β-actin (AC-15) was from Sigma. Anti-mouse-IgG and anti-rabbit-IgG were from Jackson. Immunoreactive proteins were visualized using ECL (GE Healthcare, Milwaukee, WI, USA) or Immobilon (Millipore) western blotting detection reagents. The signals on the membrane were quantified with a LAS-3000 Luminoimager (Fujifilm, Tokyo, Japan).

### *In vivo* Chromatin Immunoprecipitation

Frozen livers from mice fed with either casein or β-conglycinin were crushed into powder. DNA-protein cross-linking was performed by incubating powdered liver tissue (50 mg) with 1% formaldehyde in PBS containing 1 mM DTT and 1 mM PMSF for 10–15 min at room temperature with gentle shaking. Cross-linking reactions were stopped by adding glycine to 0.125 M. Liver nuclei were isolated with a Dounce homogenizer in hypotonic solution followed by centrifugation at 4000 × g for 1 min. Chromatin immunoprecipitation assays using liver nuclei were performed using a ChIP assay kit (Upstate Biotechnology) and anti-ATF4 antibody (5 μg, Santa Cruz Biotechnology, Inc.) or control rabbit IgG (Millipore Corporation) or anti-acetyl-Histone H3 (Lys9 [Upstate Biotechnology]). Precipitated DNA was purified using a phenol-chloroform method and eluted in 50 μl water. DNA was subjected to RT-qPCR analysis using the following oligonucleotides: *FGF21* −6533/−6470 forward: 5′-TCAGCATGCCTCCAAAGC-3′, reverse: 5′-TCAGCCTTGAGGAAGAGTAGACA-3′; *FGF21* AARE1 forward: 5′-TGACTGCAGGAAACAACCCA-3′, reverse: 5′-AGCTGCTGTCCTCCCTGAT-3′.

### RT-qPCR Analysis

Primers were designed using Primer Express software (Applied Biosystems) based on GenBank sequence data. For RT-q-PCR reactions, mice cDNA was extracted by ISOGEN (Nippon Gene Inc.) and synthesized using high capacity cDNA Reverse Transcription kit (Applied Biosystems), and qPCR was performed using SYBR Green Master Mix (Roche). All reactions were performed on Step One Plus Real-Time PCR system (Applied Biosystems), and relative mRNA levels were calculated by the comparative threshold cycle method by using cyclophilin or 18S as the internal control. Sequences of primers used in RT-qPCR analysis are listed in Supplementary Table 4.

### Generation and administration of recombinant adenoviruses

The recombinant adenoviruses were generated using the ViraPower™ Adenoviral Expression System (Invitrogen) in 293 A cells according to the manufacturer’s instructions. The viral chromosome, which contains target proteins, was linearized by PacI and transfected into 293 A to produce P1 virus. High-titer stocks of amplified recombinant adenoviruses were purified by two-step ultracentrifugation in cesium chloride gradient. After dialysis, viral titers were determined by the tissue culture infectious dose 50 method using 293 A cells. Viruses were diluted in PBS and administered through tail vein injection, using approximately 1 × 10^9^ pfu/mice.

### DNA microarray analysis

Transcriptional profiling of mouse liver was performed using the Mouse Genome 430 2.0 Array (Affymetrix, Inc., USA) and a 3′IVT Express kit (Affymetrix, Inc., USA), according to the Affymetrix protocols (Affymetrix Genechip Fluidics Workstation). Briefly, 100 ng of total RNA was reverse transcribed into cDNA using a poly (dT) oligonucleotide attached to a T7 promoter and converted to dsDNA. This was then used as a template for the synthesis of biotinylated aRNA by T7 RNA polymerase. Labeled aRNA was fragmented and hybridized to the array at 45 °C for 16 h. After hybridization, the array was washed and stained with streptavidin–phycoerythrin. Fluorescent signals were scanned using the Affymetrix GeneChip System.

### Analysis of DNA microarray data

Affymetrix GeneChip Command Console software was used to reduce the array images to the intensity of each probe (CEL files). CEL files were quantified using the Factor Analysis for Distribution Free Weighted method (dfw) algorithm using the statistical language R. Differentially expressed genes were identified by applying the Rank Products method to the dfw quantified data. The annotation file for the Mouse Genome 430 2.0 Array was downloaded from the Affymetrix website. DAVID was used to detect overrepresented GO terms in each group of DEGs. We compared the DEGs with the Mouse Genome 430 2.0 Array background. The functional annotation chart, an integrated tool in DAVID, was applied to examine significantly overrepresented GO terms. A modified fisher exact *P* value (Expression Analysis Systematic Explorer score) of <0.05 was accepted as significant.

### Statistical Analysis

The data are expressed as mean ± SD. Statistical analysis was performed using the Ekuseru-Toukei Ver.2.0 (Social Survey Research Information). Comparisons between treatments were made by a Student’s *t test* for two groups. One-way ANOVA followed by the Bonferroni procedure was used to compare more than two groups. Differences were considered significant at *P* < 0.05.

## Additional Information

**How to cite this article**: Hashidume, T. *et al*. Single ingestion of soy β-conglycinin induces increased postprandial circulating FGF21 levels exerting beneficial health effects. *Sci. Rep.* **6**, 28183; doi: 10.1038/srep28183 (2016).

## Supplementary Material

Supplementary Information
