# Supplementary material for: Single ingestion of soy β-conglycinin induces increased postprandial circulating FGF21 levels exerting beneficial health effects
Source: Sci Rep. 2016 Jun 17;6:28183. doi: 10.1038/srep28183 (PMC4911586; doi:10.1038/srep28183)
Supplement: Supplementary Information [file srep28183-s1.doc]

**Supplementary Information**

Single ingestion of soy β-conglycinin induces increased postprandial circulating FGF21 levels exerting beneficial health effects

**Tsutomu Hashidume^1,2^, Asuka Kato^1^, Tomohiro Tanaka^1^, Shoko Miyoshi^1^, Nobuyuki Itoh^3^, Rieko Nakata^4^, Hiroyasu Inoue^4^, Akira Oikawa^5^, Yuji Nakai^6^, Makoto Shimizu^1^, Jun Inoue^1^,Ryuichiro Sato^1*^**

From the

^1^Department of Biotechnology, Graduate School of Agricultural and Life Sciences, The University of Tokyo, Tokyo 113-8657, Japan

^2^Institute of Gerontology, The University of Tokyo, Tokyo 113-8656, Japan

^3^Medical Innovation Center, Kyoto University Graduate School of Medicine, Sakyo, Kyoto 606-8507, Japan

^4^Department of Food Science and Nutrition, Nara Women's University, Kita-Uoya-Nishi-Machi, Nara, 630- 8506, Japan

^5^RIKEN Center for Sustainable Resource Science, Yokohama, Kanagawa, Japan; Faculty of Agriculture, Yamagata University, Tsuruoka-shi, Yamagata 997-8555, Japan.

^6^Institute for Food Science, Hirosaki University, Aomori 038-0012, Japan

*Address for reprint requests and other correspondence: R. Sato, 1-1-1 Yayoi, Bunkyo, Tokyo 113-8657, Japan (e-mail: [aroysato@mail.ecc.u-tokyo.ac.jp](mailto:aroysato@mail.ecc.u-tokyo.ac.jp)).

Supplementary Table1. Amino acid composition of experimental diets

Supplementary Table2. Amino acid concentrations in the culture medium for mouse primary hepatocytes

Supplementary Table3. Composition of experimental diets

Supplementary Table4. PCR primers used in this study

Supplementary Figure1. **Western blot analysis of hepatic regulatory proteins after ingestion of the indicated diets.**

Five-week old male mice were acclimated to the casein high-fat diet for 3 days. After 24 h fasting, mice were fed with 1.2 g of one of the indicated high-fat diets (Supplementary Tables 1 and 3), and then their livers were obtained at 1 or 2 h after the beginning of feeding. Protein was extracted from samples using cell lysis buffer. Membranes were probed with the anti-ATF4 (Cell Signaling Technology), anti-phospho-eIF2α (Cell Signaling Technology), anti-eIF2α antibody (Cell Signaling Technology), or anti-β-actin antibody (Sigma). The same results were obtained in two separate experiments.

Supplementary Figure2. **Western blot analysis of PGC-1a in subcutaneous adipose tissue of wild-type (WT) and FGF21-deficient mice.**

Five-week old male WT or *Fgf21*-deficient (FGF21KO) mice were acclimated to the casein high-fat diet for 3 days. After 24 h fasting, mice were fed with either the casein or β-conglycinin (β-con) high-fat diet for 2 h. Protein was extracted from subcutaneous adipose tissue using cell lysis buffer. Membranes were probed with the anti-PGC-1α (Sigma) and anti-β-actin antibody (Sigma). The same results were obtained in two separate experiments.

Supplementary Figure3. **Changes in energy expenditure in mice fed either the casein or** **β-conglycinin diet for 5 days.**

Five-week-old male WT mice were acclimated to the casein high-fat diet for 7 days, and then fed either the casein or β-conglycinin (β-con) diet for 9 weeks. Open-circuit indirect calorimetry was performed for 5 days with an O_2_/CO_2_ metabolism measuring system ARCO-2000 for small animals (ARCO SYSTEM Inc., Chiba, Japan). Mice were acclimated in the metabolic chambers for 24 h and then had free access to food and water for the subsequent 5 days in a 12-h light (ZT 0–12)/dark (ZT 12–24) cycle. The system monitored VO_2_ and VCO_2_ at 1-min intervals and calculated the respiratory quotient (RQ) ratio (VCO_2_/VO_2_), and locomotor activity of mice. Each value from individual time points were combined and the data normalized for body weight were analyzed by unpaired Student’s *t* test (n = 4).

Supplementary Figure 4. **Serum adiponectin levels of wild-type (WT) and FGF21-deficient (FGF21KO) mice fed either the casein or β-conglycinin diet.**

Serum was obtained as shown in Figure 2. Serum Adiponectin concentrations were measured using Mouse Adiponectin Quantikine ELISA kit (R&D Systems, Inc.). All data are expressed as means ± SD (n = 6-8).  The same results were obtained using three different types of ELISA kits.

**Supplementary References**

1. Saito, T., Kohno, M., Tsumura, K., Kugimiya, W. & Kito, M. Novel method using phytase for separating soybean beta-conglycinin and glycinin. *Biosci. Biotechnol. Biochem.* **65,** 884–7 (2001).
